# Perceptions of E-Cigarettes among Black Youth in California

**DOI:** 10.3390/ijerph14010060

**Published:** 2017-01-11

**Authors:** Catherine A. Hess, Tamar M. J. Antin, Rachelle Annechino, Geoffrey Hunt

**Affiliations:** 1 Prevention Research Institute, Pacific Institute for Research and Evaluation, 180 Grand Avenue., Suite 1200, Oakland, CA 94612, USA; tantin@prev.org (T.M.J.A.); rannechino@prev.org (R.A.); 2 Institute for Scientific Analysis, 1150 Ballena Blvd., Suite 211, Alameda, CA 94501, USA; gh.crf@psy.au.dk; 3 Center for Alcohol and Drug Research, Aarhus University, Bartholins Allé 10, Building 1322, 334, 8000 Aarhus C, Denmark

**Keywords:** ENDS, Black youth, cultural commodity, identity

## Abstract

Research suggests that Black youth are less likely to use e-cigarettes than their white counterparts, yet little is known as to why. We examined perceptions of e-cigarettes among Black young adults (ages 18–25) to explore the meanings these youth ascribe to e-cigarettes and the role that identity plays in how these devices are viewed. Analysis of in-depth interviews with 36 Black smokers and non-smokers in the San Francisco Bay Area suggests that Black youth perceive e-cigarettes as serving distinct, yet overlapping roles: a utilitarian function, in that they are recognized as legitimate smoking cessation tools, and a social function, insofar as they serve to mark social identity, specifically a social identity from which our participants disassociated. Participants described e-cigarette users in highly racialized and classed terms and generally expressed disinterest in using e-cigarettes, due in part perhaps to the fact that use of these devices would signal alignment with a middle class, hipster identity. This analysis is discussed within a highly charged political and public health debate about the benefits and harms associated with e-cigarette use.

## 1. Introduction

Use of electronic nicotine delivery systems (ENDS), e.g., e-cigarettes, is increasing among youth in the United States [1]. While the devices are purportedly marketed to adult smokers as alternatives to cigarettes or as smoking cessation devices, research suggests that young people with no history of cigarette use are increasingly experimenting with e-cigarettes. The first measure of e-cigarette use among U.S. adolescents was undertaken in the National Youth Tobacco Survey (NYTS) (2011–2012). At that time, 3% of surveyed high school students had used e-cigarettes [2]. More recent research suggests that the number of adolescents and young people using e-cigarettes has increased [3]. For example, data collected from NYTS 2015 suggests that approximately 16% of all high school students in the United States currently use e-cigarettes [4]. The public health implications of this increase are currently a subject of debate [5,6,7]. 

Though e-cigarettes appear to be increasing in popularity among youth and young adults generally, e-cigarette initiation and use nevertheless varies across youth groups. Similar to patterns of conventional cigarette use [8], Black adolescents may initiate e-cigarette use later and be less likely to continue using e-cigarettes compared to their White or Hispanic counterparts [4,9]. For example, Lippert et al. (2015) surveyed over 15,000 6th through 12th graders in the U.S. and found that Black students were significantly less likely to have ever used e-cigarettes compared to White students [10]. In addition, data from the NYTS 2015 suggests that only 8.9% of Black high school students used e-cigarettes in the past 30 days e-cigarettes compared to 17.2% of non-Hispanic White students.

Similar trends are present among young adults. McMillen and colleagues (2015) conducted nationally representative surveys each year between 2010 and 2013. Among young adults aged 18 to 24, current e-cigarette use increased in prevalence from 0% to 14% during this time [1]. Among Black young adults, however, only modest increases in the prevalence of e-cigarette use were observed, suggesting less adoption of e-cigarettes among Black youth compared to White or “Other” ethnic youth. Lower prevalence of e-cigarette use among Black youth raises questions about how Black youth perceive of these devices, yet, to date, the research is unclear. For example, Wackowski and colleagues (2016) found that though a majority of young adults perceive e-cigarettes as less harmful than cigarettes, the same is not true for Black young adults [5]. Conversely, Peters and colleagues (2016) surveyed 47 teenage boys in the southern U.S. who currently use e-cigarettes, approximately half of whom were Black [11]. Analysis of the focus group data suggested a widespread belief among participants, regardless of ethnicity, that e-cigarettes were healthier than cigarettes [11]. 

From a public health perspective, lower e-cigarette uptake among Black youth may be regarded positively or negatively [12]. Though the biomedical literature argues that any nicotine is problematic for young people due to issues related to brain development and long-term nicotine dependency, alternatively social scientists have raised critical questions about the degree to which e-cigarettes play an important role in tobacco harm reduction [12,13]. From this point of view, lower levels of e-cigarette uptake among cigarette smokers may represent more, not fewer, deaths from tobacco-related illnesses than would have occurred with increased e-cigarette uptake among those who might otherwise have smoked cigarettes, though early empirical evidence for this is mixed [14,15,16]. Consequently, questions may be raised about the rejection of these devices among groups who have a high prevalence of smoking, such as low-income Black adults. Since most people who smoke initiate in adolescence or young adulthood [17], understanding nicotine and tobacco use from young people’s perspectives is important for developing prevention and policy efforts that are effective in reducing addiction and the overall prevalence of smoking [17,18,19]. To begin to explore the relatively low levels of adoption of e-cigarettes among Black youth and young adults, we sought to gain a greater understanding of the ways in which these youth perceive e-cigarettes, with a focus on exploring why some choose to use or not use these devices.

### Conceptual Framework

To understand how Black youth perceive e-cigarettes, we draw on social science theories regarding social and cultural identity and the use of commodities to communicate those identities [20]. Nearly all goods signal some social information, whether that be the reinforcement of cultural boundaries, social status, kinship or belonging [21,22]. As such, e-cigarettes are not solely physiologically or psychologically important, but their use is also tied to the construction of social identities that differentiate youth cultures. 

Cultural attitudes towards products are shaped by the degree to which products are associated with utilitarian functions (e.g., an alternative to smoking or as a way to relieve stress), as well as social identity functions (e.g., indicating group membership) [23]. In this way, consumer products take on a social signaling role, with the use of a given product signaling values and characteristics of the user, including attributes of the user’s social identity, to others [23]. As Douglas and Isherwood argue, “Commodities are good for thinking: treat them as a non-verbal medium for the human creative faculty” [20] (p. 40). 

Individuals may also choose not to use a product, or actively avoid certain objects or behaviors in order to disassociate with social groups [23]. In this case, the decision not to use a specific product or engage in a particular behavior also serves to communicate identity [23,24]. Berger and Rand (2008) proffer a simple anecdotal example of dissociative signaling in a survey of undergraduate students at Stanford University, who collectively spurn bicycle helmets despite the numerous accidents that occur on campus each year and the known protective benefits of helmet-wearing [25]. Undergraduates cited a strong desire not to be mistaken for graduate students who do tend to wear helmets. In this scenario, choosing not to wear a helmet served as a social signal that the cyclist was not a graduate student, and the signaling function of helmet-wearing, or *non*-helmet-wearing, superseded the unequivocally positive utilitarian function [25]. Thus, dissociative signaling can have a powerful influence on individual behavior, making products more or less attractive or accessible to different youth groups. In this paper, we are concerned with understanding the ways in which e-cigarettes are socially positioned as cultural commodities, facilitating their use for some and their rejection for others.

## 2. Methodology

### 2.1. Research Design

Between April 2014 and July 2015, we conducted multiple method in-depth interviews with 46 Black (ages 18–25) smokers and non-smokers in the San Francisco Bay Area for a study designed to understand perceptions of tobacco control policies and the intersections of tobacco-related stigma with other social identity stigmas (e.g., race, gender, socio-economic status). Of the 46 participants in the study, 36 participants discussed these devices in their interviews, and 15 had used e-cigarettes at some point in the past.

Participants were recruited on the street, through Facebook and Craigslist advertising, and by referrals. The interview guide was informed by (1) existing qualitative research on smoking among youth as well as by (2) exploratory focus groups we conducted with Black youth prior to data collection in an effort to identify potentially salient issues [26]. E-cigarette questions and probes were included for exploration, since this topic was not the main purpose of the study. Given the lack of literature on e-cigarettes among youth of color, an exploratory investigation was appropriate [27]. The interview guide resulted in closed-ended questions to collect basic demographic information about nicotine and tobacco use, and open-ended questions about the background of the participant, racial identity, personal tobacco and e-cigarette use, smoking identity, beliefs about tobacco, the social acceptability of tobacco, and perceptions and use of e-cigarettes. Interviews lasted approximately two and a half hours and were digitally recorded. To show our appreciation for their time, participants received a $40 honorarium. All study procedures were approved by the Pacific Institute for Research and Evaluation Institutional Review Board (The PIRE IRB project number is 987961-2).

### 2.2. Analysis

Following the interviews, the interviewer completed field notes to record preliminary analytical ideas emergent from interviews and to contextualize interviews with descriptive information not captured in audio recordings. Interviews were digitally recorded and professionally transcribed. Two research staff coded transcripts to distill data into manageable analytical segments, using ATLAS.ti (ATLAS.ti Scientific Software Development GmbH, Berlin, Germany), a qualitative data management system. During the coding process, researchers wrote memos to capture their analytical thinking about the data. Memos serve to contextualize coded narrative data with researchers’ emergent interpretations that are based on the entire context of the interview. The code list was extensive to capture main study objectives, but for the purposes of the present analysis, only one relevant code was included (i.e., ENDS) which indexed any narrative in interviews that either referenced e-cigarettes directly or was relevant to understanding perceptions of e-cigarettes. After the coding process, the lead author conducted a second more directed yet exploratory pattern-level analysis of all narrative segments coded as ENDS and all related memos. Patterns of emergent themes were identified primarily by examining the frequency and omission of topics across interviews [28]. Frequency refers to a theme emerging as particularly salient across all interviews, while omission is defined by themes identified in the literature as salient yet surprisingly absent in the empirical data for the study. This process revealed rich insight into the meaning and perceived role of e-cigarettes for study participants. Pseudonyms, selected by participants, are used throughout the findings to identify quotations.

## 3. Findings

Analysis of the patterns of participants’ narratives illustrated that Black youth in this study perceive e-cigarettes as serving distinct, yet overlapping roles: a utilitarian function, in that they are recognized as legitimate smoking cessation tools, and a social function, insofar as they serve to mark social identity, specifically a social identity from which our participants disassociated. The majority of participants in this study were of low socio-economic status, with a household income of less than $35,000 USD, technically classified as living in poverty in the San Francisco Bay Area. Twenty-four young women and 22 young men comprised the sample. Twenty-five participants were current tobacco or nicotine users defined by past 30-day use. Among the 15 ever e-cigarette users, 12 had used e-cigarettes in the previous year, and among these, all reported smoking cigarettes in the previous 30 days. Among the 36 respondents whose interviews included discussion of e-cigarettes, 21 were current smokers (10 men, 11 women) and 15 were non-smokers (five men, 10 women).

### 3.1. Social Identity Function of ENDS

Overall, participants in our study described the stereotypical e-cigarette user as someone racially, socio-economically, and/or socially different from themselves. Participants emphasized that e-cigarettes “weren’t for them,” ascribing social meanings to e-cigarette users from which participants in our study sought to disassociate. For example, “Z”, a 24-year-old non-smoking man, who has not used e-cigarettes, identified a “typical” e-cigarette user as:

“A higher-income person, (someone) who goes to the doctor and feels guilty about smoking cigarettes and tobacco, and is into technology and (chuckles) maybe rides a bike.”

The perception of a typical e-cigarette user as someone who is affluent was common among our participants, as was the concept of e-cigarettes being popular among “hipsters” and “techies” as is alluded to in the quote above and reinforced in other participants’ narratives. For example, “Jazzy”, an 18-year-old non-smoking woman, who does not use e-cigarettes, succinctly positioned e-cigarettes as a marker of social identity when describing them as products for people who are trying to look cool or trendy:

“I feel like a lot of hipsters use e-cigarettes. A hipster is someone who tries to fit in with a certain image because it looks cool. E-cigs are very trendy so they’re like ‘Ooh I look so cool with my e-cig.’”

Although hipster culture is notoriously difficult to define [29,30], it is generally characterized by a rejection of mainstream social norms, including through the adoption of smoking cigarettes, which are broadly stigmatized by society and therefore well situated to communicate hipster sensibilities. In their ethnographic exploration of hipster subculture, Maly and Varis note that “commodification and consumption is intrinsically connected to being a hipster,” since hipsters use consumer products to seek an “authenticity” that is defined in opposition to “mainstream” culture [29] (p. 4). Thus hipsters are avatars of the “very trendy”, adopting new products and behaviors in a continual effort to evade mainstream co-optation. The demographics of hipster cultures primarily include white, urban, middle class individuals who are generally between the ages of 25–35 [29]. As such, hipster cultures have long been criticized for lacking class and racial and ethnic diversity [30]. Like these critiques about hipsters in general, narratives about who uses e-cigarettes were highly racialized and classed. For example, “Diane”, a 22-year-old non-smoker who does not use e-cigarettes, explained that e-cigarette users are:

“…non-Black people… So similarly with the cigarette smoking, actually having a cigarette, that is mostly White people. Then secondary, non-Black people, non-White people. But with the e-cigarettes, I suppose it would just be non-Blacks. I’ve seen Asians smoking as well.”

“Jane Doe”, a 24-year-old woman who smokes, also stated that e-cigarettes are not something that she sees in the Black community:

“I sure don’t see Black people smoking them… I see Black people smoking real cigarettes. I never see Black people with e-cigarettes or vapors…” She considered trying e-cigarettes but did not.

Meanwhile “Dee”, a 24-year-old woman who smokes, but does not use e-cigarettes, highlighted socioeconomic factors that may contribute to the association of e-cigarettes with the “commodification and consumption (that) is intrinsically connected to being a hipster” [29].

“Who do I see smoking e-cigarettes? Upper class individuals. I haven’t seen a person of color smoking those e-cigarettes ‘cause they’re kind of expensive.’”

She went on to state that: “Usually I see affluent people smoking those… I don’t think they sell those in marginalized communities…”

“Keeno”, a non-smoking, non-e-cigarette-using man aged 24, reinforces the notion that e-cigarettes signal membership in an external group (“tech show” participants) typically associated with a hipster style of consumption. Noting increasing e-cigarette use in Oakland and San Francisco, Keeno observes:

“Let’s just be straightforward. Particularly, I’ve seen them in the hands of European Americans, white folks… I worked this tech show last week in San Francisco and I saw people with it.”

From a public health perspective, the disassociation of e-cigarettes among Black youth who smoke may be problematic due to the potential health benefits of switching from conventional smoking to e-cigarettes. One participant, “Lee”, a non-smoking man aged 21, commented that he hoped e-cigarettes would become more than just a subcultural phenomenon, raising the possibility of harm reduction through e-cigarette use for Black youth who smoke. Lee had tried e-cigarettes in the past, but did not like them, and only occasionally “takes a few puffs” from friends’ devices.

“I see a lot more business people with vape pens nowadays taking a… break outside instead of cigarettes. So that’s a good thing, in my opinion… Maybe young black kids will prefer those over… cigarettes at some point in time.”

Lee’s comment points to how e-cigarettes can be positively perceived for their utilitarian functions but still rejected when they are socially positioned as cultural commodities that signal membership in a group from which a particular youth group may be disassociated. 

### 3.2. Utilitarian Function of E-Cigarettes

In addition to the social identity signaling of e-cigarette use, analysis of the narrative data suggests that e-cigarettes were perceived to serve important utilitarian functions for some people—i.e., for smoking reduction or cessation or for minimizing cravings when smoking is prohibited. According to “Lee”, above, e-cigarettes are targeted “towards an older audience, you know, people that are trying to quit smoking or that are looking for an alternative for whatever reason”. “Black Tar Rebellion”, for example, a 21-year-old non-smoking woman, summarizes the typical e-cigarette user as: “Somebody who wants to quit or somebody who doesn’t want to smell like cigarettes.” Black Tar Rebellion, who has never smoked, briefly used a nicotine-free vape pen as a way to look rebellious, but gave it up after experiencing negative health effects. 

Participants generally regarded people who used e-cigarettes for their utilitarian function as opposed to their social identity function more positively, and their perceptions of the typical utilitarian user did not tend to carry connotations of race and class. “Jazzy”, for example, who mocked the typical e-cigarette user as a hipster who just wants to look “cool”, acknowledged a utilitarian role of e-cigarettes and stated that she has more respect for people who use e-cigarettes to quit smoking, as opposed to adopting a new trend:

“Some people who actually had a habit of smoking cigarettes, who are trying to be better, they use them. I respect them way more.”

Though participants in this study tended to disassociate from vapers and vaping, 15 participants nevertheless had experimented with e-cigarettes. In general, their initial motivation for trying the devices stemmed from health concerns about smoking, and they had heard that e-cigarettes might help to facilitate cessation. Notably, however, there was skepticism about the effectiveness of e-cigarettes as cessation tools, and not just among the participants who had tried the devices. For example, “Karoline”, a non-smoking woman aged 19, believed that e-cigarettes were primarily for individuals who cannot quit smoking, though she questioned the extent to which they work.

“People who smoke e-cigarettes are trying to get off cigarettes and they just can’t go cold-turkey, so that’s like their in-between… But it’s very ineffective on that in-between to get you off of it.”

Similarly, “Jazzy” described an acquaintance who used e-cigarettes to quit smoking, but who was ultimately unsuccessful.

“He had an e-cig, ‘cause my mom bought it for him I think‘ cause she wanted to help him. She was happy that he was trying to be better, so in order to help him on his journey, she got him some e-cigs. He would charge it. Everything was cool. He stopped using cigarettes. Now he’s just using his e-cig. Then after that, the next step would have been, ‘Okay, now you’re not even using an e-cig and you’re good. You’re fine. You don’t even need it.’… He ended up relapsing.”

In addition to skepticism about the effectiveness of e-cigarettes for smoking cessation, participants in this study who smoked were generally not interested in switching to e-cigarettes due to questions about their safety. For example, “Marley”, a 22-year-old smoker who had tried e-cigarettes to quit smoking, believes that e-cigarettes are just as harmful as cigarettes.

“It’s just weird that they say it’s not smoke, but then I learned that they about have the same, like, bad stuff as cigarettes. I thought it was healthier. That’s why I was doing it. But there’s no difference.”

Similarly, “Jade,” a 22-year-old smoker, questioned the point of switching from cigarettes to e-cigarettes: “This makes me wanna know a lot more about e-cigarettes, like what’s in them, why it’s okay to switch to e-cigarettes and it’s okay to maintain that habit, but not regular tobacco cigarettes. So I wanna know what the differences are, but then also, why is the solution to stopping one habit starting another…”

Notably, however, among the few participants who reported regular use of e-cigarettes was “Nefarious,” a 21-year-old woman who was using e-cigarettes to cut down on smoking and to minimize what she perceives to be negative consequences of smoking.

“I smoke like two cigarettes a day. But I’ll try the vape pen most of the time. Like today, I forgot my vape pen, so I’m like, damn it, I’m gonna have to go get a cigarette. I try to do as much of the vape pen as I can.”

She goes on to say that she originally purchased a vaping device because of:

“The smell (of cigarettes). That’s why I got the vape pen… I don’t smell like anything. It’s all sweet and fruity. I still smoke like two cigarettes, but I’ve been working since I was homeless to not smell like cigarettes. I was smelling like straight up cigarettes and God knows what else when I was homeless. I did not like that. My friends didn’t like it.”

Here Nefarious’ use of e-cigarettes in combination with cigarettes can be described as “dual use,” about which researchers in public health have expressed great concern due to the potential of dual use to exacerbate nicotine addiction and reinforce smoking [31,32,33]. However, Nefarious’s dual use of cigarettes and e-cigarettes illustrates her attempt to minimize the social harms associated with smoking and the ability of e-cigarettes to negate those harms. Attempting to avoid the stigma associated with smoking, in terms of smell as well as other perceived negative stereotypes of smokers, is not uncommon, particularly for people who experience multiple stigmas, including homelessness such as in Nefarious’ experience [34,35].

Few participants had adopted positions similar to Nefarious, however. Instead, participants in this study generally expressed safety concerns and/or skepticism about the effectiveness of e-cigarettes for cessation and few participants were using the devices. Research by Camenga and colleagues, who conducted focus groups with middle school, high school, and college students, also found that adolescents and young adults were skeptical about the efficacy of e-cigarettes in successful smoking cessation. Nevertheless, their participants generally perceived e-cigarettes to be less harmful than cigarettes [31]. Conversely, participants in our study tended to believe e-cigarettes were “just as harmful” as smoking, a belief reinforced by contemporary health media messages [36]. 

## 4. Limitations

Findings should be considered in light of the following limitations. Importantly, this exploratory analysis is based on the narratives of 36 Black women and men who were selected purposively for a study on perceptions of tobacco control strategies and experiences with multiple stigmas. Narrative surrounding e-cigarettes emerged occasionally throughout interviews, and after conducting 10 interviews for the study, we integrated a few open-ended questions about perceptions of e-cigarettes. As such, this study was not designed around understanding perceptions of e-cigarettes among Black youth, and, therefore, findings should be considered in light of this important limitation. Additionally, the study is limited in scope by the relatively small, non-random sample. Although this study is not necessarily a representative sample of Black youth in the San Francisco Bay Area, we did attempt to recruit a diverse range of participants (e.g., race/ethnicity, gender, geography, social class) by using a multi-tiered recruitment strategy to reach participants from a variety of social contexts and networks. Notably, qualitative research does not aim for traditional conceptualizations of generalizability or representativeness. Instead, it aims to maximize the variation of perspectives among participants to develop theory that can be tested in future large-scale studies. A final limitation to note here is that the theoretical ideas discussed in this paper are based on our research team’s interpretation of participants’ narratives, and multiple interpretations of these narratives are possible. However, we integrate standard procedures into our study designs in an effort to reduce threats to the valid interpretation of data and enhance reliability to justify analytical generalizability, i.e., the likelihood that the theoretical contribution from our study is applicable to another situation. 

## 5. Discussion

As Douglas and Isherwood wrote, “Goods that minister to physical needs… are no less carriers of meaning than ballet or poetry” [20] (p. 49). This assertion is supported by our study which illustrates how e-cigarettes, which are perceived by participants to be highly utilitarian, also are defined by the meanings they communicate to others when consumed. 

In particular, study participants offered clear individuating descriptions of a typical e-cigarette user, such as “hipster” and “rides a bike”. There is evidence of a hipster association with e-cigarettes in the literature. Tobacco companies have actively courted hipsters [37], and the same appears to be true of marketers of ENDS devices, with the makers of several popular brands of e-cigarettes incorporating elements of hipster subculture in advertisements, including retro tropes such as traditional cocktails, clothing, and craft beer [37]. E-cigarette advertising overtly highlights the hip non-conformity and intrinsic coolness of vaping. Health, 2001 describes coolness as the “central status hierarchy in contemporary urban society” [38]. However, what is “cool” is largely determined by cultural affinity, and even within a given culture, what is cool for one group may not be cool for another.

Inasmuch as the use of e-cigarettes signals group membership, *non*-use of e-cigarettes also communicates meaning about social identities. It may be that our participants express disinterest in e-cigarettes for several reasons, but arguably an underlying context of this disassociation is related to social identity. We theorize that the rejection of e-cigarettes among our study participants who smoke is, in part, a function of dissociative signaling from a white, middle class, hipster social identity. Using e-cigarettes, even for their utilitarian function, would nevertheless signal membership in a racial, social, and socioeconomic status group that does not represent the identities of participants.

Research in consumer psychology has explored why consumers will refuse to use or cease to use a product when it is associated with an unfavorable group [25,39]. For example, White and Dahl (2006) posit that individuals will avoid products associated with an out group in order to reaffirm their own group status. They argue that men, for example, tend to avoid or entirely refuse products commonly viewed as “feminine”, which reaffirms their own masculinity and group solidarity [39]. Returning to Berger and Rand’s (2008) survey of helmet wearing, dissociative signaling can become problematic when the device or commodity in question has the potential to improve the health or well-being of the user [25]. 

In our study, participants clearly viewed e-cigarettes as a tool that could aid in smoking cessation. However, study participants who smoked, some of whom indicated an interest in quitting or reducing their smoking, nevertheless expressed disinterest in e-cigarettes due to their association with other youth cultural groups. As “Lee” points out above, e-cigarettes may be inaccessible to some Black youth, which may harm them in the long run. This situation is problematic from a range of harm reductionist viewpoints, which may incorporate claims that continue to be a source of controversy among public health advocates (for example, that e-cigarettes can help smokers to quit [16,40]), as well as claims that are increasingly accepted across a range of public health perspectives to varying degrees. To what extent may the very rejection of e-cigarettes among Black youth who smoke work to further perpetuate tobacco-related health inequity? It is well established that despite initiating cigarette smoking later and smoking fewer cigarettes per day than their White counterparts, Black smokers have lower rates of smoking cessation and a younger average age of onset of lung cancer [41]. Black men, in particular, have a higher incidence of and mortality from tobacco-related cancers compared to White men [42]. Since combustible tobacco use is believed to cause a considerably higher risk of cancer than e-cigarette use, it is possible that the rejection of ENDS by young Black smokers could be an important barrier to decreasing mortality in this population [43]. 

## 6. Conclusions

Whether or not e-cigarettes are a viable long-term strategy for tobacco-related harm reduction has yet to be determined [40]. Some public health organizations outside of the U.S. are actively encouraging smokers to switch to e-cigarettes as a step towards cessation or for harm reduction, whereas public health agencies in the U.S. tended to reject this approach. There is clear conflict within the international public health community as to the role of e-cigarettes for smoking cessation with recent meta-analyses calling onto question the long-term efficacy [44,45]. However, as the population-level prevalence of smoking remains low and significant inequities in smoking and related illnesses persist, we have to prioritize alternative approaches that work to reduce tobacco-related inequities, particularly among populations such as Blacks, with disproportionately high prevalence of tobacco-related disease. Some critical scholars have argued that the e-cigarette has the potential to serve as a disruptive innovation and eradicate smoking entirely [12,46,47].

Though we are more cautious in our interpretation, we do acknowledge that studies among adults suggest that e-cigarettes can be an effective smoking cessation tool, and the majority of adult users are current or former smokers [48,49]. Studies among youth have found that there is a low prevalence of e-cigarette use among non-smoking youth, and that youth who do smoke view e-cigarettes as a less harmful alternative [50]. In our study, participants expressed disinterest in e-cigarettes for a variety of reasons, most notably because they project a social image that is unacceptable to them. However, these young adults did recognize the potential for e-cigarettes to serve as a smoking cessation tool, but concerns were expressed about their potential safety and/or effectiveness. If evidence accumulates that e-cigarettes are significantly less harmful than smoking and are effective in smoking cessation, then our findings suggesting that Black youth who smoke are disinterested in switching from smoking to vaping are especially problematic in terms of health equity.
